# Mapping Soil Salinity/Sodicity by using Landsat OLI Imagery and PLSR Algorithm over Semiarid West Jilin Province, China

**DOI:** 10.3390/s18041048

**Published:** 2018-03-31

**Authors:** Hao Yu, Mingyue Liu, Baojia Du, Zongming Wang, Liangjun Hu, Bai Zhang

**Affiliations:** 1 Northeast Institute of Geography and Agroecology, Chinese Academy of Sciences, Key Laboratory of Wetland Ecology and Environment, Changchun 130102, China; yuhao@iga.ac.cn (H.Y.); mingyueliu@iga.ac.cn (M.L.); dubaojiajl@163.com (B.D.); 2 University of Chinese Academy of Sciences, Beijing 100049, China; 3 Northeast Normal University, Key Laboratory for Vegetation Ecology Science of Ministry of Education, Changchun 130021, China

**Keywords:** soil salinity, soil sodicity, Partial Least Square Regression (PLSR), hybridized salinity and sodicity (HSS), Landsat 8 OLI

## Abstract

Soil salinity and sodicity can significantly reduce the value and the productivity of affected lands, posing degradation, and threats to sustainable development of natural resources on earth. This research attempted to map soil salinity/sodicity via disentangling the relationships between Landsat 8 Operational Land Imager (OLI) imagery and in-situ measurements (EC, pH) over the west Jilin of China. We established the retrieval models for soil salinity and sodicity using Partial Least Square Regression (PLSR). Spatial distribution of the soils that were subjected to hybridized salinity and sodicity (HSS) was obtained by overlay analysis using maps of soil salinity and sodicity in geographical information system (GIS) environment. We analyzed the severity and occurring sizes of soil salinity, sodicity, and HSS with regard to specified soil types and land cover. Results indicated that the models’ accuracy was improved by combining the reflectance bands and spectral indices that were mathematically transformed. Therefore, our results stipulated that the OLI imagery and PLSR method applied to mapping soil salinity and sodicity in the region. The mapping results revealed that the areas of soil salinity, sodicity, and HSS were 1.61 × 10^6^ hm^2^, 1.46 × 10^6^ hm^2^, and 1.36 × 10^6^ hm^2^, respectively. Also, the occurring area of moderate and intensive sodicity was larger than that of salinity. This research may underpin efficiently mapping regional salinity/sodicity occurrences, understanding the linkages between spectral reflectance and ground measurements of soil salinity and sodicity, and provide tools for soil salinity monitoring and the sustainable utilization of land resources.

## 1. Introduction

Basically, mapping and assessing the soil salinity and sodicity is a first step for salinity monitoring, salt-soil agriculture development, and environmental sustainability maintenance throughout the world [1,2]. Such work is also valuable to the scientific understanding of soil salinity and sodicity occurrences and pursuing effective measures for evaluating the affected soils [3]. 

The west Jilin Province of China, typically a semiarid area, is among the world’s three largest sodic saline-alkali regions. Due to overgrazing and grassland conversion to farmland, its area of salt-affecting lands increased drastically during the 1950s and the 1990s [4,5]. Consequently, soil salinity and sodicity decreased the productivity of the croplands and affected diversity of the grassland plant species, which thwarted ecological sustainability and societal and economic development of the west Jilin Province. For better understanding the occurring extent and severity of soil salinity and sodicity, depicting accurate projection of future salinity dynamics and making decisions about land management and utilization in the region, mapping the local salinity, and sodicity is much needed [6]. 

Soil salinity refers to the amount of soluble salt conserved in the soils, whereas sodicity points to the sodium as often means poor soil structures that impede soil hydraulic conductance and water uptake of plants [1,7]. As soil salinity and sodicity aggravates, more salt ions appear at the soil surface, which favors the use of conventional remote sensing (RS) technology for surveillance [8]. In the last decades, multispectral and microwave RS data with varied spatio-temporal resolutions (e.g., Landsat, SPOT XS, IKONOS, ASTER, MODIS, IRS, and Radar, etc.) have been used for monitoring [9,10,11] and mapping [12,13] salt-affected soils and halophytic vegetation [3,14]. In most cases, multiple bands were transformed into one index that was more sensitive to soil salinity than using a single band [15,16]. As for the single band, reflectance at the visible (VIS) bands (400~750 nm) was found to be more sensitive to soil pH than in other spectral ranges; Landsat 8 OLI-band1 (coastal) was also found closely correlated with soil salinity [17]. Nonetheless, substantial elements in linking RS spectral information and field salinity measurements are still to be elucidated, particularly across diverse regions. In particular, soil salinity was more often mentioned than sodicity in previous studies [18,19]. Yet, discerning the disparity effects between salinity and sodicity could underpin full-view recognition of soil health and making decisions about land restoration, and improve the comprehensive utilization of the lands. But, this critical issue should be fully examined especially in reference to the soils with complicated coexistence of soil salinity and sodicity in the west Jilin Province. 

The Partial Least Squares Regression (PLSR) has been widely used in soil parameter estimation [3]. This technique is a robust multivariate regression method appropriate particularly when the predictors exhibit multicollinearity [20]. Accordingly, many studies reported success of using PLSR for soil salinity assessment [21,22,23]. By contrast, Janik et al. [24] and Pang et al. [25] implied that the ANN (Artificial Neutral Network) method could outperform the basic PLSR for prediction of some soil properties and non-linear regression may be more accurate than linear regression. However, ANN is difficult to accurately analyze the various performance indices of a biblical network. Therefore, we chose PLSR to establish the inversion models of soil salinity and soil sodicity. Another consideration to improve the accuracy of modelling was that we tried to transform spectral reflectivity through non-linear functions before modelling. Here, the advantages of using the PLSR approach rely on its inference capabilities for modelling the probable linear relationships that are existing between spectral reflectance and salt content in soils, and power to include multiple response variables simultaneously for addressing the likely collinearity among the noise-engaged predictor variables. 

Accordingly, the objectives of this research were as follows: (1) to build up the relationships between reflectance bands, spectral indices (vegetation indices and salinity/sodicity indices), and field measurements of soil salinity/sodicity; (2) to improve soil salinity/sodicity estimating accuracy by optimizing PLSR; (3) to map soil salinity/sodicity of the study area, particularly the HSS; and, (4) to unravel the occurring severity and distribution of soil salinity, soil sodicity, and HSS with regard to land cover and soil types. This research may provide a clue for pursuing tools about soil salinity monitoring, understanding correlations between spectral reflectance and field measurements of salinity, as well as tackling regional land degradation and development issues. 

## 2. Materials and Methods

### 2.1. Study Area Descriptions

**Figure 1:**
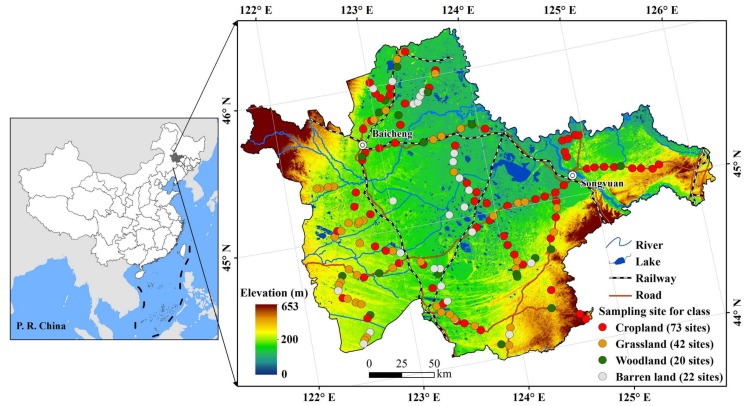
Location of the study area, digital elevation model (DEM) and soil sampling locations.

The west Jilin Province, as the largest sodic saline-alkali soil distribution in Northeast China, is located in the southern Songnen Plains (43°59′~46°18′ N, 121°38′~126°11′ E. Figure 1), with low-lying terrains and elevations between 100~200 m. The groundwater depth is about 2 m. The study area is under a temperate, semi-arid continental monsoon climate, with precipitation dwindling from 420~460 mm in the east to 350~420 mm in the west, and evaporation increasing from 1200~1600 mm in the east to 1500~2000 mm in the west [26,27]. About 80% of the total precipitation occurs between mid-June and mid-August. The soils are Chernozem (Haplic Chernozem, FAO), meadow soil (Eutric Vertisol, FAO), aeolian soil (Arenosol, FAO), Solonetz (Solonetz, FAO), and Chestnut soil (Haplic Kastanozem, FAO). Pristine vegetation of this area is temperate steppes grown on the salted soils, with widespread fragmented scar-like saline lands that were spotted on the landscapes. However, grassland conversion to cropland has been common since the 1980s in this region, leading to less than one-quarter of the natural steppes are merely remained and cultivated croplands are ubiquitous with salinity to some extent.

### 2.2. Data Sources and Pre-Processing 

Six scenes of satellite images created by Landsat 8 OLI covering the study region were used, of which five scenes were acquired on August 2016, and one image at the end of July. The images were geo-rectified to the Universal Transverse Mercator (UTM) coordinate system using World Geodetic System (WGS) 1984 datum assigned to north UTM Zone 51. Atmospheric correction was performed using FLAASH Model (Fast Line-of-sight Atmospheric Analysis of Spectral Hypercubes) that can correct both additive and multiplicative atmospheric effects [28]. Using ENVI 5.3, the study region was clipped from a mosaic of all the processed images. The spectral indices that link vegetation performance and soil salinity were calculated by the reflectance of corresponding bands, including NDVI, EVI, SIs [2,15,29], DVI [30], SAVI, and SRSI [31]. The spectral indices are listed in Table 1.

For enhancing the remotely sensed data that built the linkages between spectral signatures and soil salinity and verifying the inversion models on the retrieval of soil salinity and sodicity, we conducted field measurements and soil sampling in October 2016. As a result, all of the soil samples were collected in triplicate from the 0–5 cm layer. The number of soil samples obtained from each land cover type is shown in Figure 1. All of the samples were air-dried, sieved through a 2 mm sieve. Soil pH and EC were measured in 1:5 soil:water extracts using LEICI pH5-3E and LEICI DDS-307A meters, respectively [17]. Wetland, water body, and built-up land were not involved in the inversion because we focused on the predominantly terrestrial salinity, and thus excluded samples from the lands. By excluding four data points with aberrant values following experimental analysis, we finally accepted 157 sampling points for analysis.

Auxiliary data included Digital Elevation Model (DEM, resolution 30 m) data; a soil type map (1:1000,000), provided by Northeast Institute of Geography and Agroecology, Chinese Academy of Sciences; and, reference/validation site for classification of land cover by field survey or from Google Earth in 2016.

### 2.3. Random Forest to Classifying Land Cover

The RS images were classified by Random Forest (RF) to draw a land cover map for analyzing soil salinity and sodicity within each land cover type. RF is an ensemble of classification trees, where each tree contributes with a single vote for the assignment of the most frequent class to the input data. Key advantages of RF include non-parametric nature, high classifying accuracy, and capability to weigh variable importance [32,33]. RF uses a random subset of inputs or predictive variables in a node division instead of using best variables, which can reduce the generalizing error and finally lead to highest accuracy. Here, the classification system consisted of seven basic land cover classes: woodland, grassland, wetland, water body, barren land, cropland, and built-up [34,35]. The overall land cover classification accuracy was 95.76%, with a Kappa value 0.91 for RF. 

### 2.4. The PLSR

The PLSR is an extension of multiple regression analysis, in which the effects of linear combinations of several predictors on a response variable (or multiple response variables) are analyzed. The method combines the traits of principal component analysis and multiple linear regressions [20]. Therefore, to eliminate the influence of collinearity among variables on multiple linear regression models, PLSR is usually used to improve the stability of estimators [36]. For developing the PLSR algorithms in this research, soil EC and pH were used each as an indicator of soil salinity and sodicity. Based on field sampling, the PLSR models can be established for soil salinity and sodicity retrieval [3,16,36]. The variables were transformed into six formulas to better retrieve soil EC and pH before three times of PLSR analyses. For the first time, soil EC and pH were retrieved merely by the reflectance of bands. Subsequently, we only used spectral indices to build up the retrieval models. Finally, we combined the bands and spectral indices to retrieve soil EC and pH. By using R^2^ and RMSE, the models were assessed to determine best models. Consequently, the optimized latent variables for regression were obtained from the models for soil salinity and sodicity. Model performance can be evaluated with R^2^, Bias, Standard Deviation (SD), and RMSE. R^2^ indicates the strength of statistical correlation between measured and predicted values. The model can be accurate for R^2^ > 0.91, good for 0.82 < R^2^ < 0.90, moderate for 0.66 < R^2^ < 0.81, and poor for 0.5 < R^2^ < 0.65, according to Farifteh et al. [37]. Bias measures the mean difference between prediction and measurements, and SD represents the random component of total uncertainties [38]. The metrics are described, as follows:

(1) $$R^{2}=1-\frac{\sum {\left({\hat{y}}_{i}-y_{i}\right)}^{2}}{\sum {\left(y_{i}-\bar{y}\right)}^{2}}$$

(2) $$\mathrm{Bias}=\frac{\sum \left({\hat{y}}_{i}-y_{i}\right)}{N}$$

(3) $$\mathrm{SD}={\left[\frac{\sum {\left({\hat{y}}_{i}-y_{i}-\mathrm{Bias}\right)}^{2}}{N-1}\right]}^{1/2}$$

(4) $$\mathrm{RMSE}={\left[\frac{\sum {\left({\hat{y}}_{i}-y_{i}\right)}^{2}}{N}\right]}^{1/2}$$

where y denotes the measures with a mean value of $\bar{y}$, $\hat{y}$ denotes the predicted values, and N the number of samples. The 0.05 probability level was used to test the significance of correlation.

## 3. Results

### 3.1. Sensitivity of RS Variables to Soil EC and pH

In order to distil the variables sensitive to soil pH and EC, spectral analyses of soil samples were implemented. Regression coefficients were used to indicate the impact of independent variables on the dependent variable, for instance, the higher the regression coefficient value, the greater the impact on the dependent variable [3]. Before modelling, the 157 soil samples were divided into two subsets. The first set (110 samples) was used for calibration and the second set (47 samples) for validation. Laboratory analysis showed that the pH values of the sampled soils ranged from 5.90 to 10.45, with an average 8.70 that generally pointed to sodicity [1]. EC measurements of the samples ranged from 0.03 to 3.99 mS/cm, with an average 0.54 mS/cm that indicated lower soluble salt concentration. Thus, most soils in the study area were more affected by sodicity rather than by salinity.

Table 2 shows the correlation coefficients between EC, pH, and reflectance of bands, and between bands. EC showed the lowest coefficient positive with NIR (R = 0.348), moderately correlated with SWIR1, SWIR2, and PAN (R = 0.704, 0.738, and 0.763), and higher with Coastal, Red, Green, and Blue (R = 0.821, 0.810, 0.826, and 0.818). In particular, the correlation coefficient between Red vs. pH (R = 0.805), or between EC vs. Green (R = 0.826) was the highest each among the group, implying that the band Red had priority for soil salinity retrieval and the band Green for sodicity.

Table 3 indicates the correlation coefficients between soil EC, pH, and all of the spectral indices one another. Vegetation indices (NDVI, EVI, DVI, and SAVI) were negatively correlated with EC and pH, with NDVI/SAVI the most negatively correlated. By contrast, salinity indices (SI, SI2, SI3, and SI4) were positively correlated with EC and pH. Since SI (R = 0.803) and SAVI (R = 0.814) exhibited more sensitive to soil sodicity than other spectral indices, thus we stipulated that SAVI was the priority spectral index to reflect soil sodicity. For EC, SI3 was the best estimator of soil salinity (R = 0.826), yet EVI had the poorest (R = −0.449). Overall, the RS-derived vegetation indices were more sensitive to soil sodicity than to soil salinity. 

### 3.2. The PLSR Models for Soil Salinity/Sodicity Retrieval

We conducted mathematical transformations on all of the normalized variables, which included the bands and spectral indices before building regression models. The results implied that mathematical transformations upon the normalized variables that integrated bands and spectral indices should be carried out before building regression models, which improved the retrieval accuracy of soil salinity and sodicity than before. 

For better comparing accuracy of the inversion models after different forms of mathematical transformation, all of the variables were normalized, as follows [39]:

(5) $$x^{\prime }=\left(x-x_{min}\right)/\left(x_{max}-x_{min}\right)$$

where x is an original value, $x^{\prime }$ is the normalized value.

The variables transformed into “1/log(r)” or “e^r^” exhibited better correlations with soil EC (Table 4). For retrieval of soil pH, the “1/r” was best transform (Table 5). At the same time, we found that some nonlinear transformations can enhance the correlations between the variables and EC or pH, and the accuracy of the regression models that were established by combining bands and spectral indices was improved than that by bands or spectral indices per se. The prediction results showed that the R^2^ for EC increased from 0.704 to 0.735, and from 0.666 to 0.694 for pH. The optimized models were highlighted in Table 4 and Table 5. For the EC regression model, the most influential variables were SI3 and the band Green, which were transformed through “e^r^”. For the pH regression model, the most influential variables were SAVI and the band Coastal, which were transformed through “1/r”. Obviously, the optimized variables and the mathematical transformations were pivotal in well predicting soil EC and pH.

The prediction accuracy was assessed with 47 validating samples using RMSE and R^2^, which revealed the RMSE of the pH reaching 0.427 (R^2^ = 0.747) and that of the EC reaching 0.532 mS/cm (R^2^ = 0.698), respectively. These indicated that the two models were acceptable for estimating soil salinity and sodicity.

### 3.3. The Spatial Distributions of Soil Salinity and Sodicity 

For demonstration purpose, the retrieval results of soil EC and pH were categorized into four soil salinity levels (EC) and four sodicity levels (pH). In this research, EC values were relatively lower than some previous research [1,23,40]. It was likely due to the precipitation prior to sampling time because rainfall had a certain effect on the salinity of surface soils (0~5 cm) [41]. Thus, the salinity levels were ranked as non-salt affected (EC < 0.2 mS/cm), slightly salted (0.2 mS/cm < EC < 0.4 mS/cm), moderately salted (0.4 mS/cm < EC < 0.8 mS/cm), and intensively salted (EC > 0.8 mS/cm), following Taylor [40] and Zhang et al. [23]; and, the sodicity levels were non-sodium affected (pH < 8.5), slightly sodic (8.5 < pH < 9.0), moderately sodic (9.0 < pH < 9.5), and intensively sodic (pH > 9.5), according to Ali [1] and Farifteh et al. [42]. 

**Figure 2:**
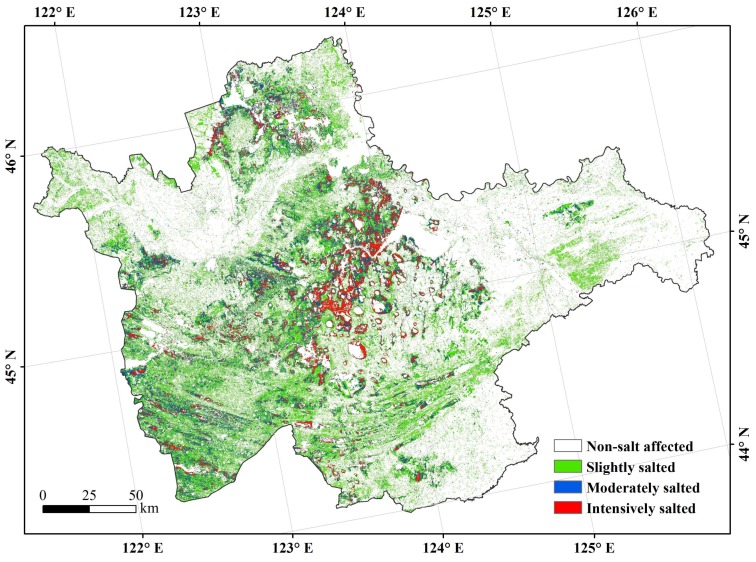
Distributions of soil salinity in the west Jilin.

The mapping results showed that the non-salt affecting area was located in northeast and southwest of the study area, and the intensively salted area was in the middle, whilst the moderately salted area was in the south and middle of the area (Figure 2). The salt-affecting area was 1.61 × 10^6^ hm^2^, accounting for 34.28% of the study area; slightly salted was common in the salt-affecting lands (1.17 × 10^6^ hm^2^), accounting for 72.90% of the area. The areas of moderately salted and intensively salted were 0.23 × 10^6^ hm^2^ and 0.21 × 10^6^ hm^2^, accounting for 14.31% and 12.79% of the salt-affecting area, respectively. 

**Figure 3:**
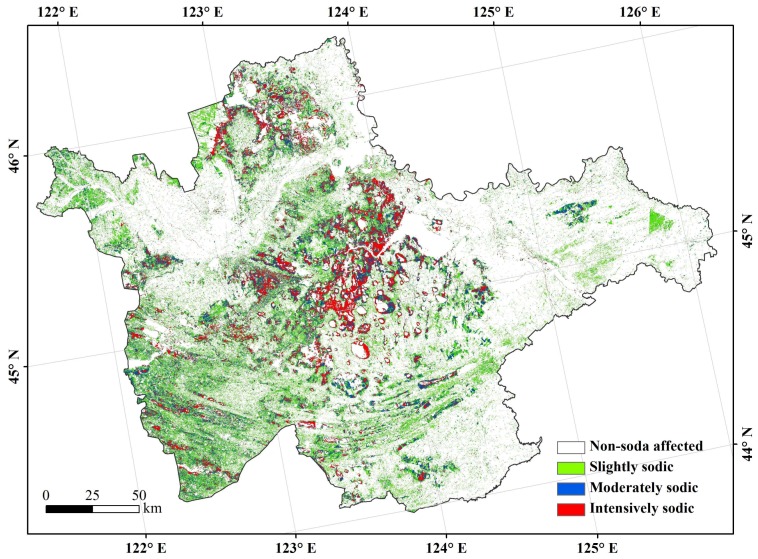
Distributions of soil sodicity in the west Jilin.

The sodium-affecting area was 1.46 × 10^6^ hm^2^, accounting for 31.15% of the study area; slightly sodic was the dominant level (0.79 × 10^6^ hm^2^), accounting for 54.51% of the area. The areas of moderately sodic and intensively sodic were 0.41 × 10^6^ hm^2^ and 0.25 × 10^6^ hm^2^, accounting for 28.14% and 17.35% in the sodium-affecting area, respectively. The intensively sodic area was distributed in the middle and the north of the study area, and moderately and slightly sodic areas were in the south and middle. By contrast, the non-sodium affecting areas were close to the non-salt affecting area, which was mainly located in the northeast and southwest of the study area (Figure 3). 

**Figure 4:**
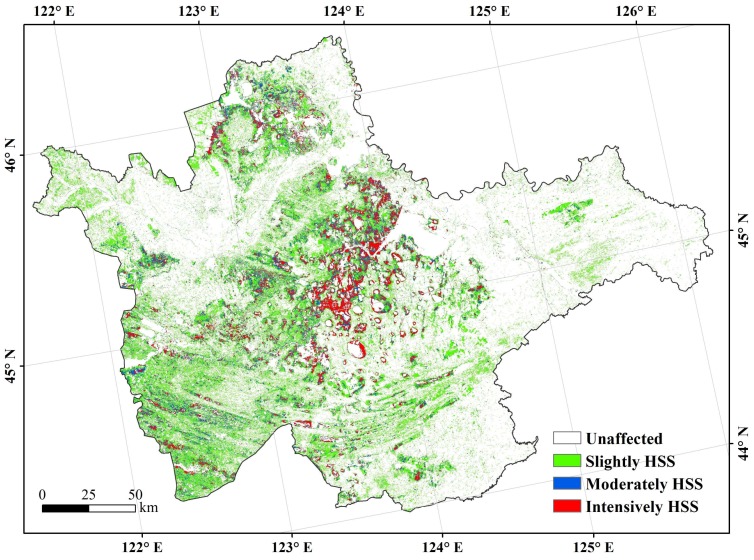
Distributions of hybridized salinity and sodicity (HSS) in the west Jilin.

Spatial distribution of the soils that were affected by HSS was obtained by overlay analysis with the maps of soil salinity and sodicity. The HSS levels are shown in Table 6. The spatial distribution of HSS indicated that the occurrence of HSS appeared roughly at the same place of soil salinity and soil sodicity (Figure 4). The HSS affecting area was 1.36 × 10^6^ hm^2^, accounting for 28.91% of the study area. The areas of moderate and intensive HSS were 0.24 × 10^6^ hm^2^ and 0.19 × 10^6^ hm^2^; accounting for 18.07% and 14.14% in the HSS affecting area, respectively. The intensive HSS area was distributed in the middle of the study area, as revealed manifest spatial overlap between the salinity and sodicity occurrences.

### 3.4. Land Cover Classifications

**Figure 5:**
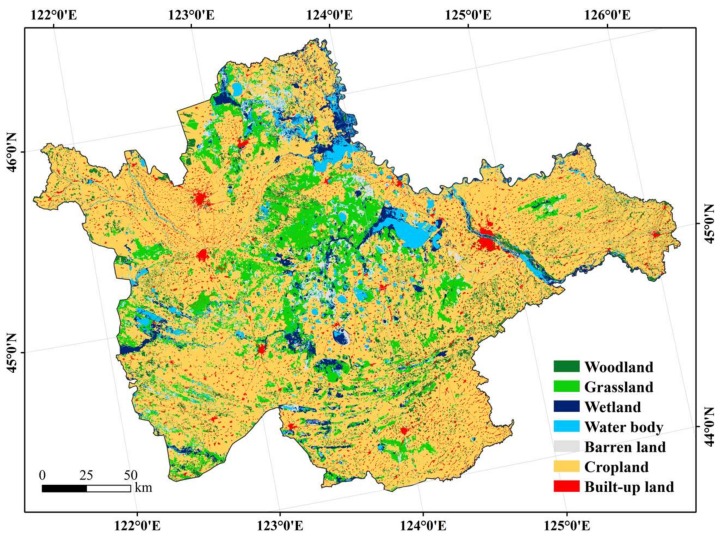
Spatial distribution of land cover in the west Jilin.

The spatial distribution of land cover in the west Jilin Province is shown in Figure 5. The results indicated that cropland was the largest land cover, with an area of 3.05 × 10^6^ hm^2^, accounting for 65.00% of the study area, as followed by grassland (12.93%), woodland (5.03%), water body (4.77%), wetland (4.64%), built-up land (4.34%), and barren land (3.29%). Grassland and barren land were mainly distributed in the middle of the study area, where many small lakes and ponds were available; woodland scattered across the area, with many as shelter belts on the fields. Accordingly, these classification results can be a basis for assessing soil salinity and sodicity in relation to land cover in the study area.

### 3.5. The Distribution of Soil Salinity/Sodicity with Land Cover

**Figure 6:**
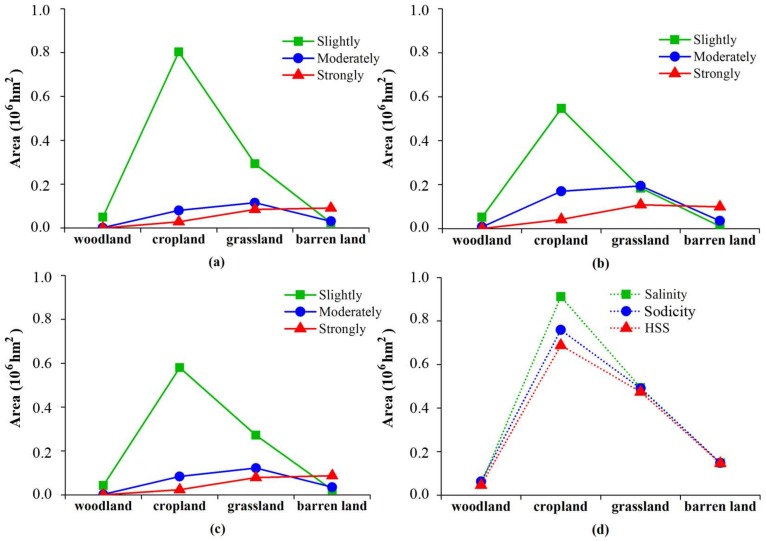
The areas of soil salinity, sodicity, and HSS at different levels with regard to land cover (**a**) Salinity (**b**) Sodicity (**c**) HSS (**d**) Affected soils.

In this research, we merely analyzed soil salinity, sodicity, and HSS for woodland, cropland, grassland, and barren land in the study area. Figure 6 illustrates the areas of soil salinity, sodicity, and HSS with regard to land cover. The results indicated that cropland was the largest in area among all of the land cover types, with the areas of soil salinity, sodicity, and HSS to be 0.91 × 10^6^ hm^2^, 0.76 × 10^6^ hm^2^, and 0.69 × 10^6^ hm^2^, respectively. Grassland, barren land, and woodland followed then. However, the slightly salted level resided the prevalent degree in the affected area among cropland, grassland, and woodland, which emerged the same for the slightly HSS level. Further, the barren land was mostly intensively salted and/or sodic (with about 60.00% of the barren land). As for grassland, moderately sodic level was dominant in the sodium-affecting area (32.24%), whereas woodland exhibited the least affected by salinity and sodicity. 

### 3.6. The Distribution of Soil Salinity/Sodicity with Soil Types

**Figure 7:**
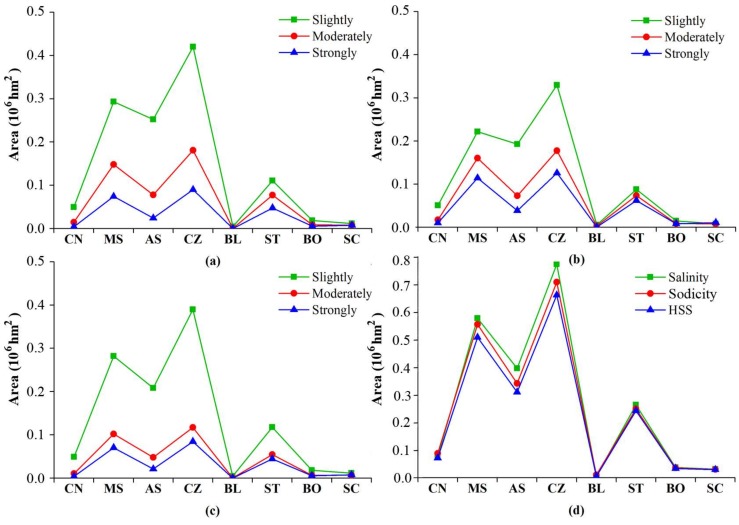
The areas affected by soil salinity, sodicity, and HSS at different levels with regard to soil types. (**a**) Salinity (**b**) Sodicity (**c**) HSS (**d**) Affected soil types (CN: Chestnut soil, MS: Meadow soil, AS: Aeolian soil, CZ: Chernozem, BL: Black soil ST: Solonetz, BO: Bog soil SC: Solonchak).

Figure 7 presents the areas of soil salinity, sodicity and HSS with regard to soil types. In this research, soil types encompassed eight classes in the west Jilin Province. Chernozem soil and Meadow soil were dominant in this area. The areas of soil salinity, sodicity, and HSS, with regard to soil types, followed as: Chernozem > Meadow soil > Aeolian soil > Solonetz > Chestnut soil > Bog soil > Solonchak > Black soil. The results stipulated that Chernozem was the largest in area among the soil types, in which the areas of soil salinity, sodicity, and HSS were 0.76 × 10^6^ hm^2^, 0.71 × 10^6^ hm^2^, and 0.65 × 10^6^ hm^2^, respectively. Meadow soil and Aeolian soil followed as the second and the third. Solonetz soil was the most seriously affected by salinity, sodicity and HSS, in which the influenced percentages in area were 72.14%, 68.34%, and 66.27%, respectively. Solonchak soil then followed, with an affected percentage of 57.72% in area. In addition, the soils with affected percentages above 35.00% in area included Aeolian soil, Meadow soil, and Bog soil. The soils with affected percentages below 35.00% in area pointed to Chestnut soil, Chernozem, and Black soil (Mollisol). Naturally, the Black soils were least affected among the soils. 

## 4. Discussion

This research endeavored to map soil salinity and sodicity via building the relationships between Landsat 8 OLI imagery and in-situ measurements of soil EC and pH over the west Jilin of China. We pursued establishing and improving the models for soil salinity and sodicity retrieval using PLSR and mathematical transformation of selected variables. As a result, we corroborated that combining Landsat 8 OLI imagery and improved PLSR algorithm can efficiently optimize the retrieval models of soil salinity (measured with soil EC) and sodicity (measured with soil pH). 

**Figure 8:**
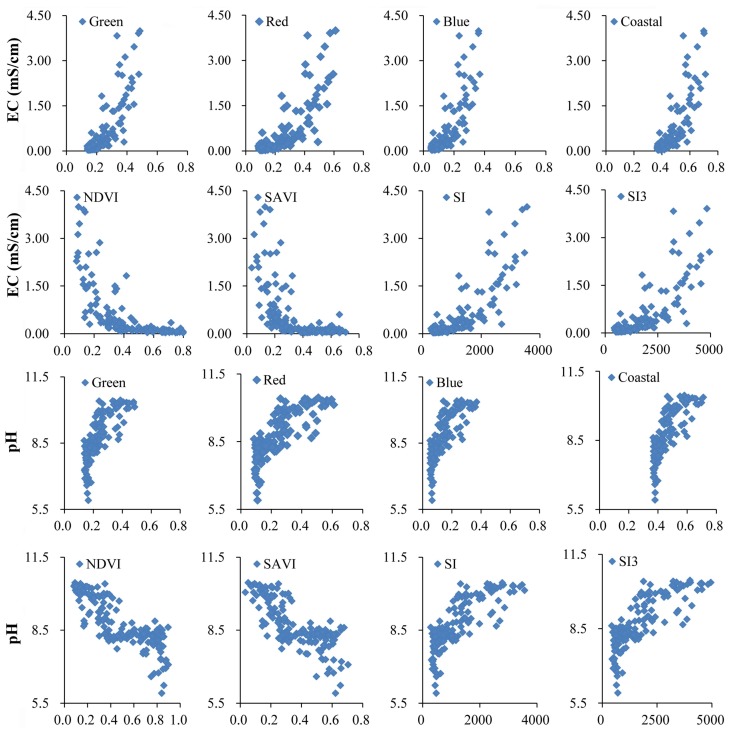
Correlations between optimal variables and soil EC and pH measurements.

In the past decades, soil salinity and/or sodicity retrieval models have been proposed often by relating soil physi-chemical properties and spectral performance with RS data and ground measurements [29,43]. By using three dielectric retrieval algorithms, small perturbation, optical physics, and Dubois models to implement analysis, Bell et al. [14] retrieved an improved estimation of a dielectric constant for soil salinity discrimination (optimized R^2^ = 0.649). Fan et al. [16] established a soil salinity retrieval model (R^2^ = 0.749) with advanced Multi-Spectral Sensor and PLSR. Janik et al. [24] documented a method by combining partial least square (PLS) and ANN (R^2^ = 0.94). Nawar et al. [36] suggested the multivariate adaptive regression splines (MARS) model (R^2^ = 0.73) for soil salinity prediction, which performed better than PLSR. Sidike et al. [3] found that PLSR was better than Stepwise Multiple Regressions (SMR) in retrieving soil salinity. However, we found that the spectral reflectance increased with soil EC, which became dramatic when EC exceeded 1 mS/cm; while pH exceeded 8, the spectral reflectance increased dramatically too (Figure 8). This implied nonlinear relationship might exist between soil EC, pH and the spectral data. Therefore, unlike previous research, we improved the PLSR models’ accuracy via combining reflectance bands and spectral indices through nonlinear mathematical transformation before modelling. Consequently, we confirmed that mathematical transformation indeed could improve the correlation between spectral reflectivity and surface sampling measurements, which further improved the accuracy of the PLSR models. Here, “e^r^”and “1/r” were the optimal mathematical transforms for building the inversion models of soil EC and pH, respectively. 

Then, we verified that Landsat 8 OLI imagery was appropriate and economic for extracting the information of soil salinity and sodicity over a large area. Generally, the advantages of RS technology involve quick access and saving time, wider coverage, and constant time series provision for long term monitoring [6,16,44]. By contrast, OLI imagery owns broader views, comparable spatial resolution relative to satellite hyperspectral data (e.g., Hyperion [45]), and abundant band/spectral information competent with higher resolution imagery like QuickBird, IKONOS, and GeoEye-1 [46]. Most importantly, Landsat 8 OLI is free to obtain [47]. 

Further, we witnessed that linking Landsat OLI-derived variables and field measurements of salinity and sodicity indeed made sense for the mapping and assessment in our case. We found that the band Green best correlated with soil EC, and the band Red best correlated with soil pH (Table 2). Yet, Bai et al. [17] found the band Coastal was best correlated with soil EC and pH. This may lie in the imagery acquisition time difference between spring season in their case and summer growing season in ours. Undoubtedly, it should be better to represent soil EC by reading vegetation performance during summer, as can be readily described by the band Green [29]. As for spectral indices, we found the vegetation indices revealed negatively, and the salinity indices were positively correlated with soil EC and pH. Moreover, the vegetation indices were more sensitive to soil pH than to soil EC, NDVI/SAVI was best correlated spectral index, and SI3 was the most sensitive salinity index to soil EC (Table 2). Nonetheless, Shrestha [11] found that NDVI was poorly negatively correlated with soil EC, and NIR was best correlated with soil EC. 

Finally, our mapping results indicated that about one third of the area was affected by salt and sodium in the west Jilin Province. The salt-affecting area was larger than that by sodicity, however, the severity of soil salinity was less than that of sodicity, because the moderate and intensive sodicity accounted for 45.49% of the sodic area, and the moderate and intensive salinity accounted for 29.03% of the saline area. Bai et al. [17] have ever estimated the soil sodicity and salinity for the northern Songnen Plains. But soil sodicity in our study was much more serious (mean pH = 8.70) than that in Bai’s study area (mean pH = 7.92). The causes may lie in less precipitation and higher temperatures in the west Jilin [26]. The soil EC values were lower when compared with other research, which may be due to the regional variations of the study areas [12,15,16,48] and field sampling uncertainties [15,38]. Despite that the soil samples were collected during a desalting period, we classified the levels of soil salinity and sodicity by expert knowledge and characteristics of the study area [14,23]. This ensures that our results can represent the spatial distributions of soil salinity and sodicity in the region well. Cropland (68.51%) was the major contributor to slight salinity, while in the moderate salinity area, grassland was dominant (50.23%). Among all of the land cover types, grassland and barren land were the major contributors to intensive salinity, with percentages 41.48% and 44.31% in area. Cropland and grassland were also dominant contributors to slight and moderate sodicity in the area. However, some differences existed between soil salinity and sodicity, e.g., the contribution rate of cropland to intensive sodicity was twice of that to intensive salinity in area.

However, several aspects should be consolidated in our research. First, we did not sample the soils from built-up land, wetland, and waters for the sake of targeting terrestrial off-town salinity issues. However, the excluded areas were also likely affected by salt problems. Second, human activities played critical roles in affecting the soil salinity and sodicity, e.g., worsening salinity and sodicity of the top soils by bringing salt to soil surface through altering natural water cycles, or allowing for excessive recharge of groundwater and salt accumulation by concentrating [1]. Built-up land is the central of human activities, however, soil salinity and sodicity in built-up land was poorly assessed [49]. Khaledian et al. [50] found that the soil quality of built-up land was poorer than that of other land types. Also, as wetland and waters are important natural resources, their salinity and sodicity will directly affect vegetation and water quality [48,51]. Therefore, to accurately analyze soil degradation by salinity and sodicity, sampling sites should be much representative, better covering all sorts of landscapes and human activities. In future research, soil samples from wetlands and urban areas are needed. Moreover, it is promising to retrospect soil salinity and sodicity during historical stages in accordance with the employed inversion models, and to unveil the areal trending of salt affection through inversion results of soil salinity and sodicity throughout multiple periods.

Land degradation, featured with land salinity mapping in our case, can be interpreted as a decline in land quality and quantity with spatio-temporal variations. However, the soils and RS data used in this study were for only one year. Hence, the use of richer time series of soils and RS data would help to boost an understanding the land degradation processes. We may focus more on the spatio-temporal dynamics of soil sodicity and salinity at diverse scales and the underlying drivers in our future investigations. 

## 5. Conclusions

We successfully mapped multiple forms of soil salinity and sodicity in the west Jilin Province of China using Landsat 8 OLI imagery and improved PLSR algorithm through the nonlinear transformation to retrieve soil salinity and sodicity. Relationships between field measurements of salinity and sodicity, reflectance bands, and spectral indices were built up; and, spatial distributions and occurring severity of salinity and sodicity were also analyzed. The results showed that the most influential variables were SI3 and the band Green were transformed through “e^r^”. For the pH regression model, the most influential variables were SAVI and the band Coastal were transformed through “1/r”. RMSE of pH reached 0.427 (R^2^ = 0.747) and that of EC reached 0.532 mS/cm (R^2^ = 0.698). 

By statistics from the mapping, about one-third of the study area was influenced by salinity and sodicity. The intensive salinity, sodicity, and HSS lands were mostly distributed in the low-elevation areas near lakes. Soil degradation due to salinity and sodicity was common with regard to land cover in the area. Barren land was the most affected land cover that was subjected to salinity and sodicity. Grassland was the most notable vegetated land cover affected by soil salinity and sodicity, in which exceeding 75.00% in area was salted. By contrast, cropland was the largest affected area among all the land cover, in which the areas that were subjected to salinity, sodicity and HSS were 0.91 × 10^6^ hm^2^, 0.76 × 10^6^ hm^2^, and 0.69 × 10^6^ hm^2^, respectively. With regard to soil types, Chernozem had the largest area subjected to salinity and sodicity, while Solonetz was the most serious type that was affected by salinity. In short, our results may provide realistic guidance for land degradation monitoring, ecological reclamation, salt-soil agriculture management, and comprehensive utilization of natural resources in the west Jilin Province of China.

## Figures and Tables

**Table 1:** The expressions of spectral indices.

Spectral Index	Expression	Full Name	Reference
NDVI	(NIR − Red)/(NIR + Red)	Normalized Differential Vegetation Index	Shrestha (2006)
DVI	NIR − Red	Differential Vegetation Index	Clevers et al. (1988)
EVI	NIR/Red	Enhanced Vegetation Index	Huete et al. (1997)
SAVI	(NIR − Red)/(NIR+Red + 0.5) × 1.5	Soil Adjusted Vegetation Index	Bouaziz et al. (2011)
SI	$\sqrt{\mathrm{Green}\;\times \;\mathrm{Red}\;}$	Salinity Index	Bouaziz et al. (2011)
SI2	$\sqrt{{\mathrm{Green}}^{2}+{\mathrm{Red}}^{2}+{\mathrm{NIR}}^{2}\;}$	Salinity Index2	Douaoui et al. (2006)
SI3	$\sqrt{{\mathrm{Green}}^{2}+{\mathrm{Red}}^{2}\;}$	Salinity Index3	Douaoui et al. (2006)
SI4	SWIR1/NIR	Salinity Index4	Douaoui et al. (2006)
SRSI	$\sqrt{{\left(\mathrm{NDVI}-1\right)}^{2}+{\mathrm{SI}}^{2}}$	Salinization Remote Sensing Index	Alhammadi et al. (2008)

**Table 2:** Correlation coefficients between band reflectance, soil pH and EC.

	EC	pH	Coastal	Red	Green	Blue	NIR	SWIR1	SWIR2	PAN
EC	1	-	-	-	-	-	-	-	-	-
pH	0.703 **	1	-	-	-	-	-	-	-	-
Coastal	0.821 **	0.791 **	1	-	-	-	-	-	-	-
Red	0.810 **	**0.805** **	0.988 **	1	-	-	-	-	-	-
Green	**0.826** **	0.793 **	0.991 **	0.992 **	1	-	-	-	-	-
Blue	0.818 **	0.795 **	0.998 **	0.992 **	0.992 **	1	-	-	-	-
NIR	0.348 **	0.068 **	0.294 **	0.245 *	0.334 **	0.283 **	1	-	-	-
SWIR1	0.704 **	0.788 **	0.912 **	0.944 **	0.925 **	0.923 **	0.160 *	1	-	-
SWIR2	0.738 **	0.791 **	0.939 **	0.967 **	0.948 **	0.948 **	0.143 *	0.989 **	1	-
PAN	0.763 **	0.773 **	0.958 **	0.964 **	0.966 **	0.963 **	0.289 **	0.906 **	0.931 **	1

Note: * represents significance at the 0.05 level; ** represents significance at the 0.01 level; bold number means the maximum value in a column.

**Table 3:** Correlation coefficients between spectral indices, soil pH and EC.

	EC	pH	SI	SI2	SI3	SI4	SRSI	SAVI	NDVI	EVI	DVI
EC	1	-	-	-	-	-	-	-	-	-	-
pH	0.703 **	1	-	-	-	-	-	-	-	-	-
SI	0.818 **	0.803 **	1	-	-	-	-	-	-	-	-
SI2	0.722 **	0.507 **	0.770 **	1	-	-	-	-	-	-	-
SI3	**0.826** **	0.793 **	1.000 **	0.804 **	1	-	-	-	-	-	-
SI4	0.472 **	0.704 **	0.731 **	0.209 *	0.695 **	1	-	-	-	-	-
SRSI	0.818 **	0.803 **	1.000 **	0.771 **	0.997 **	0.731 **	1	-	-	-	-
SAVI	−0.665 **	**−0.814** **	−0.931 **	−0.467 **	−0.888 **	−0.920 **	−0.912 **	1	-	-	-
NDVI	−0.665 **	−0.814 **	−0.931 **	−0.467 **	−0.888 **	−0.920 **	−0.912 **	1.000 **	1	-	-
EVI	−0.449 **	−0.694 **	−0.708 **	−0.159 *	−0.677 **	−0.877 **	−0.708 **	0.889 **	0.889 **	1	-
DVI	−0.555 **	−0.725 **	−0.775 **	−0.198 *	−0.736 **	−0.912 **	−0.775 **	0.935 **	0.935 **	0.909 **	1

Note: * represents significance at the 0.05 level; ** represents significance at the 0.01 level; bold number means the maximum value in a column.

**Table 4:** The regression models for soil EC in relation to bands, spectral indices and hybrid variables.

Transform	Imaging Feature	R^2^	Expression	SD	RMSE
r	Band	0.706	EC = 1.601Green − 3.093SWIR1 − 1.121	0.456	0.451
	Index	0.704	EC = 21.114NDVI + 5.411SI3 − 12.024	0.457	0.453
	Hybrid	0.706	EC = 5.854SI3 − 3.093SWIR1 − 0.029	0.455	0.451
1/r	Band	0.646	EC = − 45.758Blue − 1.031NIR + 2.670	0.501	0.527
	Index	0.707	EC = 8.506EVI − 11.121NDVI + 19.717	0.454	0.451
	Hybrid	0.712	EC = 7.915EVI + 0.161Red − 2.236	0.45	0.446
e^r^	Band	0.717	EC = 8.553Green − 1.969SWIR1 − 7.523	0.446	0.442
	Index	0.711	EC = 2.783SI3 − 3.165	0.45	0.448
	**Hybrid**	**0.735**	**EC = 14.090SI3 − 24.833Green + 13.174**	**0.432**	**0.429**
log (r)	Band	0.635	EC = 9.244Green − 3.032Red + 4.557	0.507	0.503
	Index	0.601	EC = 1.692SI3 + 2.764SI2 + 2.549	0.53	0.525
	Hybrid	0.709	EC = 20.220Green − 9.125SI + 8.066	0.452	0.449
1/log(r)	Band	0.726	EC = − 2.052Green + 0.363SWIR1 − 1.969	0.432	0.435
	Index	0.727	EC = − 0.486SI3 − 0.450	0.437	0.435
	Hybrid	0.727	EC = − 0.486SI3 − 0.450	0.437	0.435
1/e^r^	Band	0.691	EC = 4.634SWIR1 − 15.431Green + 9.478	0.467	0.462
	Index	0.691	EC = − 6.391SI3 − 7.701EVI + 3.169DVI + 8.618	0.469	0.463
	Hybrid	0.734	EC = − 90.819Green + 43.953SI3 + 38.916	0.432	0.429
Sqrt(r)	Band	0.675	EC = 11.977Green − 3.844SWIR1 − 2.951	0.479	0.474
	Index	0.682	EC = 5.083SI3 + 7.866EVI − 3.338DVI − 4.727	0.474	0.469
	Hybrid	0.724	EC = 58.351Green − 27.823SI3 − 13.214	0.441	0.437

Note: r represents the normalized variables (bands and spectral indices).

**Table 5:** The regression models for soil pH in relation to bands, spectral indices, and hybrid parameters.

Transform	Imaging Feature	R^2^	Expression	SD	RMSE
r	Band	0.666	pH = 6.082Red − 1.743NIR + 8.156	0.573	0.568
	Index	0.663	pH = − 62.762NDVI + 41.701	0.575	0.571
	Hybrid	0.686	pH = 3.753Green − 40.192NDVI + 28.970	0.557	0.551
1/r	Band	0.689	pH = − 63.919Blue − 0.499NIR + 43.330	0.554	0.548
	Index	0.691	pH = 3.541SAVI − 0.346Red − 18.863	0.552	0.547
	**Hybrid**	**0.694**	**pH = 1.705SAVI − 1.348Coastal − 1.898**	**0.551**	**0.546**
e^r^	Band	0.651	pH = 4.442Red − 1.306NIR + 5.200	0.586	0.581
	Index	0.682	pH = − 155.862SAVI + 1.049SI3 + 183.862	0.56	0.56
	Hybrid	0.684	pH = − 147.848SAVI + 2.551Green + 173.026	0.558	0.553
log (r)	Band	0.689	pH = 3.466Blue + 11.968	0.552	0.548
	Index	0.682	pH = 2.761SI + 10.582	0.558	0.554
	Hybrid	0.689	pH = 3.466Blue + 11.968	0.552	0.548
1/log(r)	Band	0.653	pH = − 2.287Blue + 0.229NIR + 6.866	0.584	0.579
	Index	0.677	pH = 493.206SAVI − 44.705NDVI + 394.719	0.564	0.559
	Hybrid	0.691	pH = 41.952SAVI − 0.559Green + 54.273	0.560	0.555
1/e^r^	Band	0.678	pH = − 8.159Red + 2.137NIR + 13.922	0.564	0.558
	Index	0.688	pH = 129.434SAVI − 3.412SRSI − 102.821	0.554	0.549
	Hybrid	0.688	pH = 129.434SAVI − 3.412SRSI − 102.821	0.554	0.549
Sqrt(r)	Band	0.678	pH = 6.139Red + 5.851	0.561	0.558
	Index	0.681	pH = 4.617SRSI + 6.529	0.558	0.555
	Hybrid	0.689	pH = 4.718SRSI − 1.647NIR + 7.643	0.553	0.548

Note: r represents the normalized variables (bands and spectral indices).

**Table 6:** The standard for soil HSS severity ranking.

Level	Range of EC and pH
Unaffected	pH < 8.5 or EC < 0.2 mS/cm
Slightly affected	0.2 mS/cm < EC < 0.4 mS/cm and 8.5 < pH < 9.0; 0.4 mS/cm < EC < 0.8 mS/cm and 8.5 < pH < 9.0; 0.2 mS/cm < EC < 0.4 mS/cm and 9.0 < pH < 9.5;
Moderately affected	0.4 mS/cm < EC < 0.8 mS/cm and9.0 < pH < 9.5; EC > 0.8 mS/cm and 8.5 < pH < 9.0; 0.2 mS/cm < EC < 0.4 mS/cm and pH > 9.5;
Intensively affected	0.8 mS/cm < EC and pH > 9.5; 0.4 mS/cm < EC < 0.8 mS/cm and pH > 9.5; 0.8 mS/cm < EC and 9.0 < pH < 9.5;
